# Oxytocin neurons in the paraventricular nucleus of the hypothalamus circuit-dependently regulates social behavior, which malfunctions in BTBR mouse model of autism

**DOI:** 10.21203/rs.3.rs-2621359/v1

**Published:** 2023-03-01

**Authors:** Hiroyuki Arakawa, Yuki Higuchi, Akihiko Ozawa

**Affiliations:** University of Ryukyus; University of the Ryukyus; Florida Atlantic University

## Abstract

Oxytocin (OXT) a neuropeptide synthesized in the hypothalamic nuclei has a variety of function including socio-emotional processes in mammals. While the neural circuits and signaling pathways in central OXT converge in the paraventricular nucleus of the hypothalamus (PVN), we illuminate specific function of discrete PVN OXT circuits, which connect to the medial amygdala (MeA) and the bed nucleus of the stria terminalis (BnST) in mouse models. The OXT^PVN^→^BnST^ projections are innervated from entire portions of the PVN, while those OXT^PVN^→^MeA^ projections are asymmetrically innervated from the posterior portion of the PVN. Compared with OXT neurons in B6 wild type mice, BTBR mice that are recognized as a behavior-based autism model exhibited defect in the OXT^PVN^→^BnST^ projection. We demonstrate that chemogenetic activation of OXT^PVN^→^MeA^ circuit enhances anxiety-like behavior and facilitates social approach behavior, while activation of OXT^PVN^→^BnST^ circuit suppresses anxiety-like behavior along with inhibiting social approach. This chemogenetic manipulation on the OXT^PVN^→^BnST^ circuit proves ineffective in BTBR mice. Accordingly, chemogenetic activation of OXT^PVN^ neurons that stimulate both OXT circuits induces OXT receptor expressions in both MeA and BnST as with those by social encounter in B6 mice. The induction of OXT receptor genes in the BnST was not observed in BTBR mice. These data support the hypothesis that OXT circuits serve as a regulator for OXT signaling in PVN to control socio-emotional approach/avoidance behavior, and a defect of OXT^PVN^→^BnST^ circuit contributes to autism-like social phenotypes in BTBR mice.

## Introduction

Social interaction is a fundamental behavior in all animal species for sustaining adaptive life and well-being. Engaging in positive social (i.e., prosocial) interactions robustly benefits health across the lifespan [1–3]. The disruption of prosocial relationships has emerged as a major symptom in many psychiatric diseases and neurodegenerative diseases [4–7]. Oxytocin (OXT) is a neuropeptide involved in a key mechanism that regulates prosocial behavior, as evidenced in several different species, including humans [8–10]. Central OXT synthesis occurs in the paraventricular nucleus (PVN) and the supraoptic nucleus (SON) of the hypothalamus and in the accessory magnocellular nuclei of the hypothalamus, such as strio- and septo-hypothalamic nuclei [10–11]. The central OXT is mainly transported via axonal projections from the PVN, distributed to various brain regions, including the basal ganglia area, such as nucleus accumbens, septal nucleus, bed nucleus of the stria terminalis (BnST), medial amygdala (MeA), and paraventricular thalamic nucleus, and mid brain areas including the raphe nucleus and periaqueductal gray [12–13]. In particular, the OXT^PVN^→^MeA^ projections are required for the processing prosocial approach behavior [14–16], while those OXT^PVN^→^BnST^ contribute to social avoidance by circuit-specific OXT action [17].

The PVN is a complex, multifunctional and multitransmitter nucleus, which modulate many CNS processes, including stress-related hormonal regulation [18]; thus, either social interaction or exploration of unfamiliar objects induces neural activation in the PVN [19]. Activation of the PVN neurons by these stimulus exposures also follows OXT release and OXT-ergic circuit-specific processing, which would be determined by the type and intensity of stimuli exposed. Abnormalities in either the OXT peptide or its receptor have been associated with social deficits that are prevalent in many psychiatric and neurodevelopmental disorders, including autism spectrum disorders (ASD) [20–21]. While whole-brain (e.g., intranasal) treatment with OXT failed to affect prosocial behavior in mice [22] and alleviate ASD symptoms in clinical trials [23], circuit-specific processing of OXT via the complex PVN network underlies for appropriate expressions of prosocial behaviors.

Mice are a highly social species that provide an exceptional tool for elucidating the neural mechanisms underlying prosocial behavior and its deficits [24]. Multiple lines of evidence have indicated that BTBR T + Itpr3tf/J (BTBR) mouse serves as a behavior-based ASD model resembling the characteristics of human ASD, including impaired prosocial interaction and persistent, repetitive behaviors [25–26]. A decreased neural activation of the PVN in BTBR mice in response to the presence of social stimuli compared to that in control mouse strain (e.g., C57BL/6J; B6 mice) has been documented [27–29]. Altered OXT neuronal morphology in the PVN was also evident in both sexes of BTBR mice compared to B6 mice [27, 29]. We hypothesized that (1) OXT signaling in the PVN is relayed via circuit-specific processes to control appropriate expression of prosocial behavior, and that (2) mal-formation or disconnection of these OXT circuits underlies social deficits exhibited by BTBR mice. Using immunohistochemical mapping of neural activity patterns and cell-type specific tracing of OXT projections, we identify modified neural activity and altered OXT projecting patterns in the PVN of BTBR mice. Our data obtained by chemogenetic manipulation of PVN neurons demonstrate that two massive OXT projections, OXT^PVN^→^MeA^ and OXT^PVN^→^BnST^, discretely regulate social and non-social behaviors, and a modification of these projections and binding receptor expressions are determinants for social deficits in BTBR mice. In summary, the data demonstrate a circuitry role for PVN OXT signaling in the expression of prosocial behavior, and reveal a possible OXT mechanism underlying deficiency in BTBR mice as an ASD mouse model.

## Materials And Methods

### Animals

BTBR T + Itpr3tf/J (BTBR) mice (stock # 002282) and C57BL/6J (B6) mice (stock # 000664) of both sexes were originally purchased from The Jackson Laboratory (Bar Harbor, ME, USA), were used as subject and stimulus mice. They were bred and maintained in the colony room of a facility at the University of the Ryukyus Faculty of Medicine, Japan. The mice were housed in groups of three or four in standard shoebox cages (28 × 22.5 × 14 cm height) with water and food provided ad libitum. The vivarium was maintained at 50% humidity and 23 ± 1°C, with a 12-h light/dark cycle (lights on at 08:00). All procedures including animal care and use were performed in line with the National Institutes of Health Guide for the Care and Use of Laboratory Animals that comply with the ARRIVE guidelines and approved by the Institutional Laboratory Animal Care and Use Committee at the University of the Ryukyus Graduate School of Medicine.

### Experimental design

For full description of methods, see Supplementary information. Briefly, we firstly measured neural activity in the PVN sub-portions (rostral to caudal) and related brain regions of B6 (n = 22) and BTBR mice (n = 21) in response to a novel social or object encounter. Then we conducted a series of behavioral tests to assess social and non-social profiles relevant to autism-like behaviors in B6 and BTBR mice. Our test battery consisted of a set of anxiety/locomotor task (the elevated plus maze), social behavior task (the modified three-chamber test), and defensive behavior task (odor defense test). These subject mice including male and female received a bilateral AAV injection for DREADD manipulation and assigned into one of five AAV conditions (Vehicle; B6, n = 24, BTBR, n = 26, ns PVN; B6, n = 22, BTBR, n = 20, OXT-PVN; B6, n = 20, BTBR n = 20, OXT-MeA; B6, n = 19, BTBR, n = 17 and OXT-BnST; B6, n = 14, BTBR, n = 14). Testing was performed in tandem with small groups of mice consisting of three-to-four cohorts of animals at different times. Following a completion of behavioral testing, AAV transduction in the targeted brain sites was histologically verified by microscopy with transduced fluorescence and immunohistochemical staining of OXT neurons. Finally, mRNA expressions of targeted genes in social processing brain regions following a social encounter or chemogenetic activation of OXT^PVN^ neurons were evaluated in B6 (n = 32) and BTBR mice (n = 30).

## Results

### PVN neuronal activity in response to social and non-social encounter

**Figure 1:**
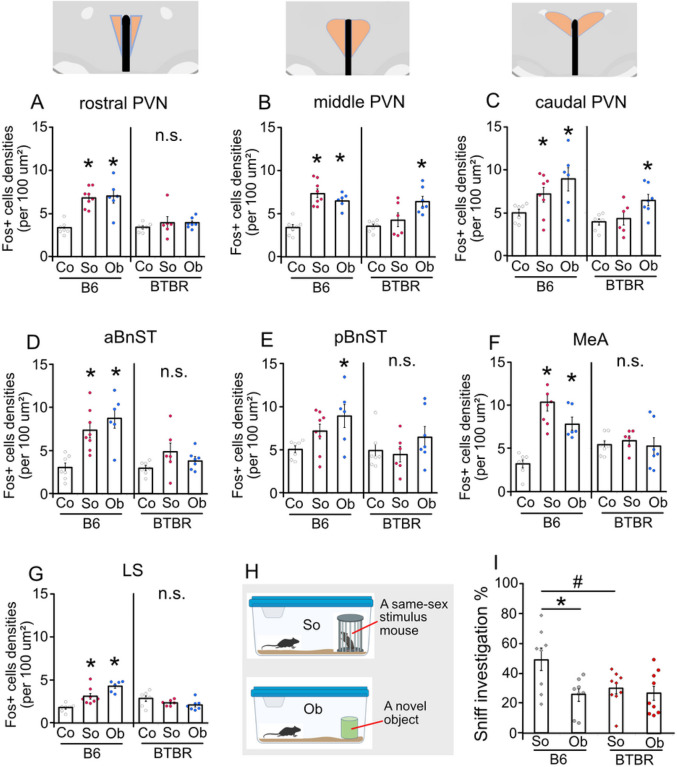
c-Fos induction is evident in B6 mice, in response to an exposure of an object (Ob) or social (So) stimuli, but blunted in BTBR in response to So exposure. Across the rostral (**A**, *F*_*2,18*_ = 1.178, *p* < 0.0001), middle (**B**, *F*_*2,18*_ = 25.13, *p* < 0.0001) to caudal (**C**, *F*_*2,18*_ = 20.94, *p* = 0.0004) PVN portions of B6 mice, induction of c-Fos is significantly increased both by object and social stimuli. The c-Fos induction is not revealed in the rostral PVN portion of BTBR mice (*F*_*2,17*_ = 0.5842, *p* = 0.5683), by either an object or social exposure, while it was increased in the middle (F_2,17_ = 7.687, *p* = 0.0042) and caudal PVN of BTBR mice (*F*_*2,17*_ = 5.5772, *p* = 0.0137), following an object, but not social exposure. In B6 mice, c-Fos induction is shown in aBnST (**D**, *F*_*2,18*_ = 11.732, *p* = 0.0005), MeA (**F**, *F*_*2,18*_ = 21.11, *p* < 0.0001), and LS (**G**, *F*_*2,18*_ = 18.787, *p* <0.0001) following either object or social stimuli, and in pBnST (**E**, *F*_*2,18*_ = 4.3158, *p* = 0.0294), only by an object exposure. However, these Fos inductions are not evident in BTBR mice in either aBnST (*F*_*2,17*_ = 2.0808, *p* = 0.156), pBnST (*F*_*2,17*_ = 1.2557, *p* = 0.31), MeA (*F*_*2,17*_ = 0.2689, *p* = 0.767), or LS (*F*_*2,17*_ = 2.4028, *p* = 0.1205), by either an object or social stimulus exposure. **H** Test conditions of stimulus exposure. **I** During the stimulus exposure, B6 and BTBR mice sniff/contact to a confronted object in similar level (p = 0.876), while BTBR mice exhibit lower sniff/contact than B6 mice to a confronted social stimulus (p = 0.0252) (2-way ANOVA, interaction of strain × stimuli, *p* = 0.056, main effect of strain, *p* = 0.0876, main effect of stimuli, *p* = 0.0127). * indicates difference between stimuli compared with homecage controls (Co), *p* < 0.05. # indicates difference between strain, *p* < 0.05. n.s., Not significant.

We firstly confirmed neural responses of the PVN sub-regions to social and non-social stimuli in B6 and BTBR mice. To map neural activity patterns in the PVN following exposure to novel social and non-social (object) stimuli, we performed immunohistochemistry for c-Fos across the rostral-caudal divides of the PVN (Fig. 1A,C) and its projecting regions; MeA (Fig. 1F), BnST (Fig. 1D,E), and LS (Fig. 1G) in B6 and BTBR mice that were exposed to a social (a novel same-sex mouse) or non-animated object, or remained in a home cage as a no exposure control (Fig. 1H)(see **Fig. S1**). For B6 mice, across the rostral to caudal portions of the PVN, significantly more cells expressing c-Fos in response to either social or non-social stimuli compared to the control condition were documented (Fig. 1A,B,C). For BTBR mice, however, the induction of c-Fos was found through the middle to caudal portions of the PVN only by an object, but not social, exposure. This effect mapped c-Fos induction disappeared in the rostral portion of the PVN. In the projecting regions from the PVN, the numbers of c-Fos + cells were increased both by social and non-social (object) exposures in B6 mice, and these were blunted in BTBR mice. Accordingly, sniffing/contact investigation towards exposed stimuli was demonstrated in both B6 and BTBR mice; however, these were attenuated in BTBR mice when confronted with a social stimulus (Fig. 1I). Therefore, a blunted c-Fos induction in targeted regions in response to social encounter exhibited by BTBR mice is covariant with decreased social investigation.

### Oxytocinergic neural projection patterns reveal strain difference

**Figure 2:**
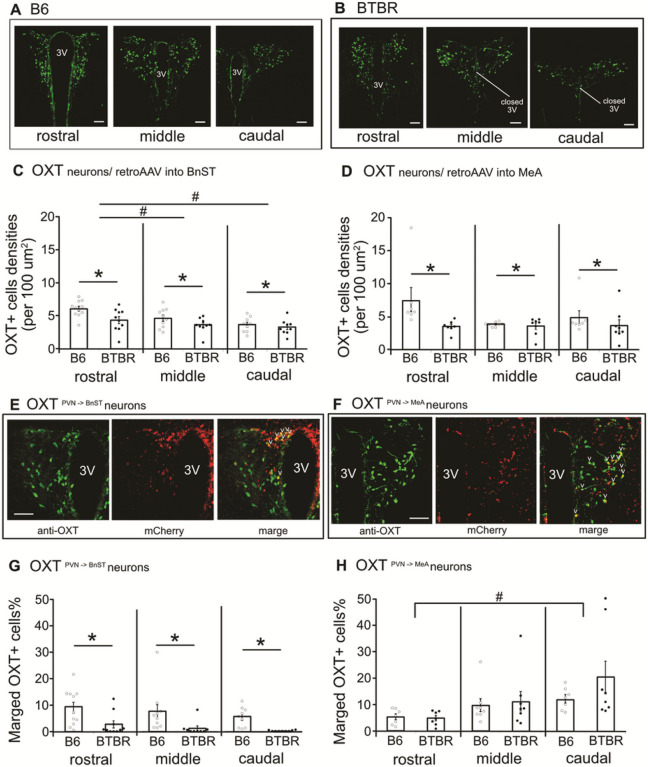
Densities of OXT+ cells are lower in BTBR mice than B6 mice across the rostral to caudal portions of the PVN. OXT^PVN→BnST^ neurons are attenuated in BTBR, while those OXT^PVN→MeA^ projecting neurons are consistently distributed both in BTBR and B6 mice. Representative immunostaining of anti-OXT antibody (green) across the rostral to caudal PVN of B6 (**A**) and BTBR (**B**) mice. **C** In both strains of mice received retroAAV (retroAAV-pOXT;mCherry) into BnST or **D** those received retroAAV into MeA, the densities of OXT+ cells are higher in B6 than BTBR mice across the PVN portions (BnST, 2-way ANOVA, interaction of strain × PVN portion, *p* = 0.298, main effect of strain, *p* = 0.0037, main effect of PVN portion, *p* = 0.00009; MeA, 2-way ANOVA, interaction of strain × PVN portion, *p* = 0.1182, main effect of strain, *p* = 0.0175, main effect of PVN portion, *p* = 0.1236). **E,F** Representative pictures depict OXT+ cells (green), OXT promoter AAV infected cells (red), and those marged (yellow) neurons in B6 mice that were injected with AAV into the BnST (OXT^PVN→BnST^ neurons; **E**) or those B6 mice received AAV injection into the MeA (OXT^PVN→MeA^ neurons; **F**). **G** OXT^PVN→BnST^ neurons are abundant in B6 mice, while those are faded in BTBR mice (2-way ANOVA, interaction of strain × PVN portion, *p* = 0.9389, main effect of strain *p* = 0.0001, main effect of PVN portion, *p* = 0.2054). **H** OXT^PVN→MeA^ neurons are expressed in both B6 and BTBR mice and more dominant its distribution in the caudal PVN portion than rostral portion (2-way ANOVA, interaction of strain × PVN portion, *p* = 0.4421, main effect of strain, *p* = 0.2942, main effect of PVN portion, *p* = 0.0098). * indicates difference between strain, *p* < 0.05. # indicates difference between PVN portion, *p* <0.05. n.s., Not significant. scale bar = 100 mm

OXT neurons of the PVN are primarily responsible for the innervation of forebrain regions [30]. While dendritic OXT release are also evident [31], OXT neurons of the PVN send axonal projections to various distances of brain regions, including the basal forebrain area, such as septal nucleus, BnST, and MeA, and several thalamic and hind brain regions [12–13]. We chose two massive OXT projecting regions, MeA and BnST, based on functional contribution to the regulation of social behavior [14, 16–17]. Immunohistchemical analysis confirmed that OXT neurons are distributed across the rostral to caudal portions of the PVN in both B6 and BTBR mice, while densities of OXT neurons are lower in BTBR mice than those in B6 mice constantly across the PVN sub-portions (Fig. 2C,D). Using a retrograde AAV vector encoding mCherry with selective OXT promoter (retroAAV-pOXT;hM3Dq-mCherry), we investigated the distribution of OXT neurons projecting to the BnST or MeA within the PVN neuronal population. OXT^PVN^→^BnST^ neurons are distributed widely across the rostral to caudal portions of the PVN in B6 mice, while these OXT^PVN^→^BnST^ neurons are present in only trace amounts in BTBR mice (Fig. 2G). Furthermore, there was no strain difference in the ratio of OXT^PVN^→^MeA^ neurons between B6 and BTBR mice (Fig. 2H). The OXT^PVN^→^MeA^ neurons tend to locate massively in the caudal portion of the PVN in both B6 and BTBR mice.

### The OXT^PVN^→^MeA^ circuits facilitate anxiety-like behavior differs from the OXT^PVN^→^BnST^ circuits

The EPM is a popular test method to measure anxiety along with locomotor activity in rodent models [32]. BTBR mice display a variable profile of anxiety-like behaviors in the EPM (e.g., increase or decrease) when compared with B6 strains [25, 33–34, 62]. In present study, both male and female BTBR mice exhibited a low anxious profile in the EPM, with an increased locomotor activity and enhanced open arm time compared with control B6 mice (**Fig. S3**). Otherwise, sex differences of the behavioral performance in BTBR strain as well as B6 strain were not consistent in this test.

**Figure 3:**
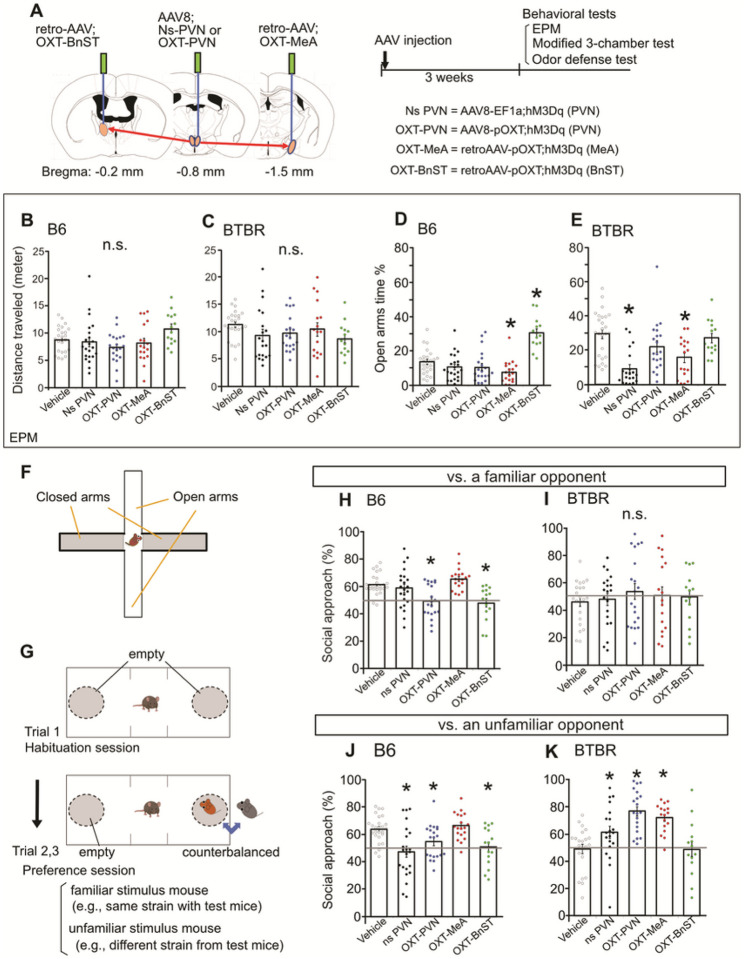
OXT^PVN→MeA^ neurons differentially regulates anxiety-like behavior in the elevated plus maze (EPM) and social approach in the modified three chamber test from those OXT^PVN→BnST^ neurons. **A** The viral strategy transfecting hM3Dq injected into the PVN, MeA, or BnST. **B,C** EPM. Chemogenetic activation of targeted neurons little impact on locomotor activity in B6 (2-way ANOVA, interaction of DREADD × sex, *p* = 0.4989, main effect of DREADD *p* = 0.0721, main effect of sex, *p* = 0.5769) or BTBR mice (2-way ANOVA, interaction of DREADD × sex, *p* = 0.3128, main effect of DREADD, *p* = 0.4303, main effect of sex, *p* = 0.7447). **D** EPM. In B6 mice, activation of OXT^PVN→MeA^ neurons decrease open-arm time % (*p* = 0.0485), while activation of those OXT^PVN→BnST^ increase open-arm time % (*p* < 0.0001)(2-way ANOVA, interaction of DREADD × sex, *p* = 0.1853, main effect of DREADD *p* < 0.0001, main effect of sex, *p* = 0.164). **E** EPM. In BTBR mice, DREADD activation of non-specific PVN neurons (*p* < 0.0001) and those of OXT^PVN→MeA^ neurons (*p* = 0.0019) decrease the open-arm time % (2-way ANOVA, interaction of DREADD × sex, *p* = 0.1728, main effect of DREADD, *p* < 0.0001, main effect of sex, *p* = 0.9219). **F** The elevated plus maze (EPM). **G** Procedure of the modified three-chamber test. **H** In B6 mice, chemogenetic activation of OXT^PVN^ neurons (*p* = 0.0007) and those of OXT^PVN→BnST^ neurons (*p* = 0.0007) suppresses approach behavior towards a familiar stimulus mouse (2-way ANOVA, interaction of DREADD × sex, *p* = 0.8039, main effect of DREADD, *p* < 0.0001, main effect of sex, *p* = 0.9429). **I** In BTBR mice, any DREADD manipulation little affect approach behavior towards a familiar mouse (2-way ANOVA, interaction of DREADD × sex, *p* = 0.5664, main effect of DREADD *p* = 0.8688, main effect of sex, *p* = 0.862). **J** When confronted with an unfamiliar mouse, chemogenetic activation of non-specific PVN neurons (*p* = 0.0003), those of OXT^PVN^ neurons (*p* = 0.0369), and OXT^PVN→BnST^ (*p* = 0.0095) induce a decreased social approach in B6 mice (2-way ANOVA, interaction of DREADD × sex, *p* = 0.9047, main effect of DREADD *p* < 0.0001, main effect of sex, *p* = 0.2178). **K** In BTBR mice, DREADD activation of non-specific PVN neurons (*p* = 0.0377), those of OXT^PVN^ neurons (*p* < 0.0001), or those of OXT^PVN→MeA^ neurons (*p* = 0.0002) increased social approach (2-way ANOVA, interaction of DREADD × sex, *p* = 0.5473, main effect of DREADD, *p* < 0.0001, main effect of sex, *p* = 0.2483). * difference between DREADD compared with vehicle controls, *p* < 0.05. # difference between strain, *p* < 0.05. + difference between sex, *p* < 0.05. n.s., Not significant.

The PVN is a center brain region for stress response [35] and anxiety behavior [36]. While OXT is known to play a modulatory role in anxiety behavior, the effects of exogeneous OXT are still variable [37] and the brain regions that transmit OXT information from the PVN for proper execution of anxiety behavior have not been identified. We applied chemogenetic manipulation on the PVN neurons to determine the cell-type and projection-specific function of the PVN circuits. In particular, we bilaterally injected AAV vector with low transduce tropism via ubiquitin promoter (AAV8-EF1a:hM3Dq; Ns PVN, labeling global PVN neurons) or OXT specific promoter (AAV8-pOXT:hM3Dq; OXT-PVN) into the PVN (Fig. 3A and **Fig. S2**). We also injected AAV vectors with retrograde tropism via OXT promoter (retro-AAV-pOXT:hM3Dq) at the MeA (OXT-MeA) and the BnST (OXT-BnST) solely, to manipulate OXT^PVN^→^MeA^ and OXT^PVN^→^BnST^ projections, respectively. In this scenario, cell expressing hM3(Dw) are categorized as various cell types in the PVN region (Ns PVN); specifically OXT neurons in the PVN (OXT-PVN); OXT^PVN^→^MeA^ projection neurons (OXT-MeA); or OXT^PVN^→^BnST^ projection neurons (OXT-BnST). This manipulation induced selective excitation of targeted neurons by an injection of a DREADD ligand, Compound 21 (C21). In the EPM, open arm time was decreased by activating OXT^PVN^→^MeA^ (OXT-MeA) but was increased when the OXT^PVN^→^BnST^ projection (OXT-BnST) was activated in B6 mice (Fig. 3D). For BTBR strain, the open arm time was decreased by activating global PVN neurons (Ns PVN) or OXT^PVN^→^MeA^ projection (OXT-MeA) (Fig. 3E). There was no impact observed on locomotor activity in both mouse strains under the current DREADD manipulation conditions (Fig. 3B,C). These data suggest that activation of OXT^PVN^→^MeA^ neurons is consistently anxiogenic in both B6 and BTBR mice, while OXT^PVN^→^BnST^ neurons is anxiolytic in B6 mice but no effect in BTBR mice.

### OXT circuits determine controlling prosocial behavior

The OXT neurons play a critical role in processing social approach and avoidance in a variety of contexts, which implies circuit-specific OXT action. The OXT^PVN^→^MeA^ neurons are responsive to facilitate social approach to conspecifics [15–16], while the OXT^PVN^→^BnST^ neurons promote social avoidance in female California mice [38] and reduce social approach in stressed mice [17]. We provide evidence that circuit-specific control of OXT in prosocial behavior is also strain-dependent.

As consonantly shown in various literatures, both male and female BTBR mice demonstrate behavioral traits relevant to autism, including reduced social approach towards familiar (c.f., same strain) or unfamiliar (c.f., different strain) social stimuli, compared to B6 controls (**Fig. S3**). Using chemogenetic manipulation, we uncovered cell-type and circuit-dependent regulation of these social behavioral traits in B6 and BTBR mice. When confronted with a same-sex familiar opponent (i.e., B6 stimulus mouse), activation of OXT^PVN^ neurons (OXT-PVN), but not those of global PVN neurons (ns PVN), decreased social approach of B6 mice (Fig. 3H). An activation of specific OXT^PVN^→^BnST^ neurons (OXT-BnST) but not OXT^PVN^→^MeA^ neurons (OXT-MeA) reduced social approach in B6 mice (Fig. 3H). However, these chemogenetic activations of PVN neurons had little impact on social approach to familiar (i.e., BTBR stimulus mouse) opponents in BTBR mice (Fig. 3I). These behavioral profiles in response to chemogenetic activation of PVN neurons differ dependent on familiarity of the opponents. When confronted with a same-sex unfamiliar mouse, activations of global PVN neurons (ns PVN), OXT^PVN^ neurons (OXT-PVN), and OXT^PVN^→^BnST^ neurons (OXT-BnST) decreased social approach in B6 mice (Fig. 3J). The social approach of BTBR mice toward an unfamiliar mouse was enhanced by activation of global PVN neurons (ns PVN), OXT^PVN^ neurons (OXT-PVN), and OXT^PVN^→^MeA^ neurons (OXT-MeA), but not by those OXT^PVN^→^BnST^ neurons (OXT-BnST)(Fig. 3K). While activation of global PVN neurons or OXT neurons in the PVN produced undirected effects on social approach in B6 and BTBR mice, selective activation of OXT^PVN^→^MeA^ neurons maintained (or enhanced) substantial level of social approach in both B6 and BTBR mice, and those of OXT^PVN^→^BnST^ neurons reduced social approach in B6 mice but maintained lower levels in BTBR mice.

### Distinct PVN circuits regulate non-social behavioral profile relevant to autism-like traits

BTBR mouse model has been associated with deficits in social communication and a pronounced engagement in repetitive behavior [25, 39]. Excessive self-grooming and persistent digging and burying the floor bedding materials (e.g., sawdust or corncob) or novel obstacles (e.g., embedded marbles) are considered to represent these later pathological states [40, 41]. The characteristics of BTBR mice suggest that variant circuitry controlling exploratory and defensive behaviors underlying their atypical behaviors needs to be elucidated. We conducted an olfactory defense test, in which mice display defensive behaviors towards exposures of neutral (ie. banana odor) and predatory (ie., fox urine compound) odor cues [42]. B6 mice displayed flexible sniff-immobile defensive patterns in tune with the type of odor stimuli, by decreased sniffing and increased immobile toward predatory aversive odor compared to those toward neutral odor (**Fig. S3E**). While BTBR mice exhibited reduced susceptibility of sniff/immobile response, they showed attenuated immobile time compared to B6 mice (**Fig. S3F**). In addition, BTBR mice exhibited a heightened levels of digging the bedding material and self-grooming compared to B6 mice (**Fig. S3G,H**).

**Figure 4:**
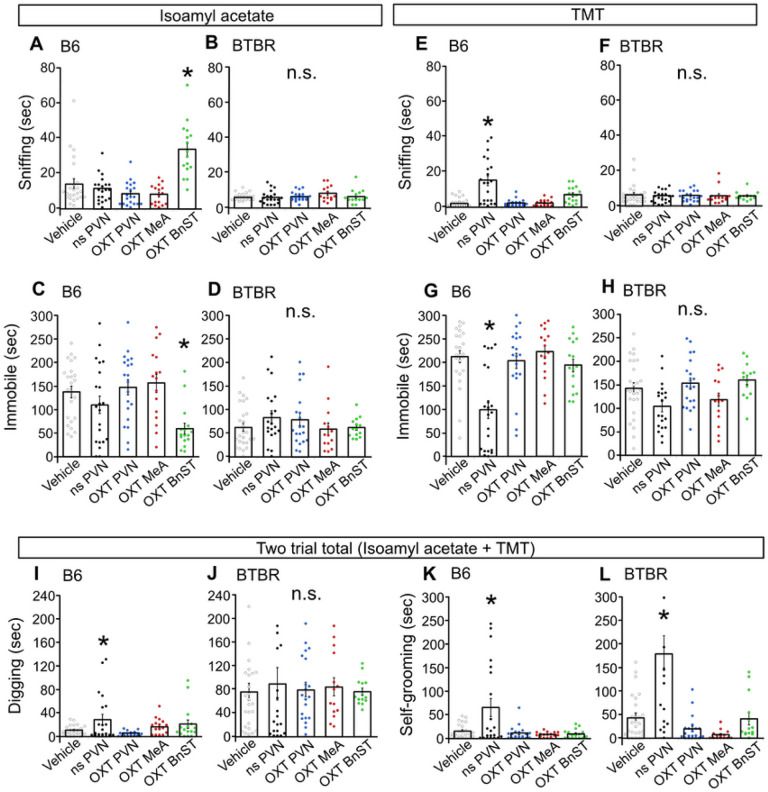
Non-OXT^PVN^ neurons are sufficient to mediate non-social, defensive behavior in both B6 and BTBR mice. **A,C** When exposed to a novel neutral odor (isoamyl acetate), chemogenetic activation of OXT^PVN→BnST^ neurons increases sniffing (*p* < 0.0001)/decreases immobile (*p* = 0.003) in B6 mice (Sniffing: 2-way ANOVA, interaction of DREADD × sex, *p* = 0.6609, main effect of DREADD, *p* < 0.0001, main effect of sex, *p* = 0.1331, Immobile: 2-way ANOVA, interaction of DREADD × sex, *p* = 0.6497, main effect of DREADD, *p* = 0.0011, main effect of sex, *p* = 0.0169). **B,D** There is no impact of DREADD on sniff/immobile towards exposure of a neutral odor in BTBR mice (Sniffing: 2-way ANOVA, interaction of DREADD × sex, *p* = 0.0411, main effect of DREADD, *p* = 0.1114, main effect of sex, *p* = 0.0018, Immobile: 2-way ANOVA, interaction of DREADD × sex, *p* = 0.8235, main effect of DREADD, *p* = 0.3805, main effect of sex, *p* = 0.547). **E,G** When confronted with a predatory odor (TMT), DREADD activation of non-specific PVN neurons increases sniffing (*p* < 0.0001) and decreases immobile (*p* < 0.0001) in B6 mice (Sniffing: 2-way ANOVA, interaction of DREADD × sex, *p* = 0.007, main effect of DREADD, *p* < 0.0001, main effect of sex, *p* = 0.0139, Immobile: 2-way ANOVA, interaction of strain × sex, *p* = 0.3804, main effect of DREADD, *p* <0.0001, main effect of sex, *p* = 0.2179). **F,H** There is no effects of DREADD manipulation on sniff/immobile towards a predatory odor in BTBR mice (Sniffing: 2-way ANOVA, interaction of DREADD × sex, *p* = 0.8902, main effect of DREADD, *p* = 0.9555, main effect of sex, *p* = 0.2298, Immobile: 2-way ANOVA, interaction of DREADD × sex, *p* = 0.675, main effect of DREADD, *p* = 0.0273, main effect of sex, *p* = 0.7122). **I,J** Chemogenetic activation of non-specific PVN neurons increases digging in B6 (*p* = 0.032) but not in BTBR (B6: 2-way ANOVA, interaction of DREADD × sex, *p* = 0.8463, main effect of DREADD *p* = 0.0209, main effect of sex, *p* = 0.6105, BTBR: 2-way ANOVA, interaction of DREADD × sex, *p* = 0.5693, main effect of DREADD, *p* = 0.9812, main effect of sex, *p* = 0.9569). **K,L** Chemogenetic activation of non-specific PVN neurons increases self-grooming in both B6 and BTBR mice (ps < 0.0001) (B6: 2-way ANOVA, interaction of DREADD × sex, *p* < 0.0001, main effect of DREADD, *p* < 0.0001, main effect of sex, *p* < 0.0001, BTBR: 2-way ANOVA, interaction of DREADD × sex, *p* < 0.0001, main effect of DREADD, *p* <0.0001, main effect of sex, *p* = 0.001). * difference between DREADD compared with vehicle controls, *p* <0.05. n.s., Not significant.

To rule out the PVN circuitry function in these defensive components, we assessed the impact of chemogenetic manipulation on these behaviors. B6 mice altered their sniff-immobile profile following an activation of OXT^PVN^→^BnST^ neurons (i.e., PVN-BnST) when confronted with a neutral natural odor (Fig. 4A,C), and following an activation of the global PVN neurons (i.e., ns PVN) when confronted with predatory odor (Fig. 4E,G). In contrast, BTBR mice did not show any change in sniff-immobile strategy towards odor stimuli via either chemogenetic PVN activations (Fig. 4F,H). Furthermore, an activation of the global PVN neurons (i.e., ns PVN) stimulated increased digging and self-grooming behavior in B6 mice (Fig. 4I,K) and self-grooming in BTBR mice (Fig. 4J,L). These data indicate that the activation of OXT^PVN^→^BnST^ neurons induces enhanced exploratory, but not defensive properties in B6 mice and this circuitry may not be responsive in BTBR mice. Global, but non OXT, PVN activation produces modification of other defensive behaviors, including immobile defense and digging and grooming behaviors, suggesting that these behavioral components are regulated by non OXT^PVN^ neurons.

### mRNA expression profiles following chemogenetic OXT^PVN^ activation and social encounter

**Figure 5:**
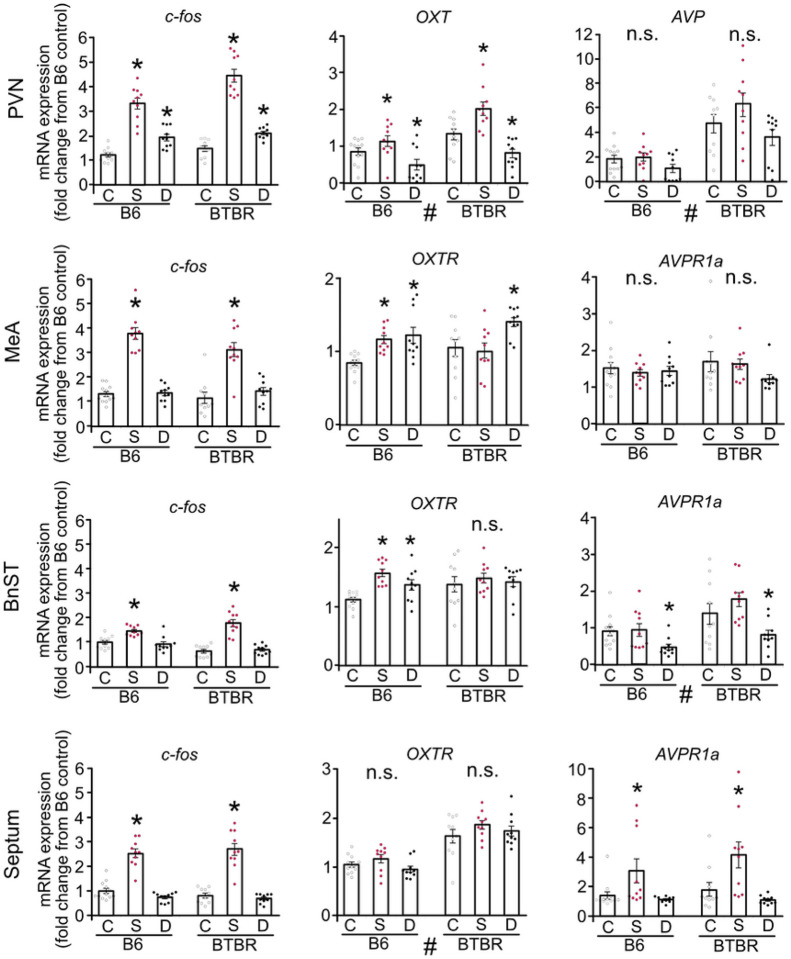
The mRNA expression of *c-fos, OXT*, and its homolog receptors (*OXTR* and *AVPR1a*) in the PVN and its projecting regions induced by social encounter (S) or chemogenetic activation of OXT^PVN^ neurons (D) in B6 and BTBR mice. In the PVN, *c-fos* expression is induced by both S and D in B6 and BTBR mice (2-way ANOVA, interaction of Strain × condition, *p* = 0.0075, main effect of strain, *p* = 0.0002, main effect of condition, *p* < 0.0001). *OXT* mRNA increases with S (*p* < 0.0001) and decreases with D (*p* = 0.0204) in both B6 and BTBR mice (2-way ANOVA, interaction of strain × condition, *p* = 0.6724, main effect of strain, *p* < 0.0001, main effect of condition, *p* = 0.0003). AVP induction is more in BTBR mice than B6 mice (2-way ANOVA, interaction of strain × condition, *p* = 0.3261, main effect of strain, *p* < 0.0001, main effect of condition, *p* = 0.0161). In the MeA, *c-fos* expression is abundant by S (*p* < 0.0001) but not by D (p = 0.4605) (2-way ANOVA, interaction of strain × condition, *p* = 0.175, main effect of strain, *p* = 0.1234, main effect of condition, *p* < 0.0001). The *OXTR* mRNA increases with S (*p* = 0.0048) and D (*p* = 0.002), in B6 mice, while it increases with D (*p* = 0.0223), but not with S (*p* = 0.7965) in BTBR mice (2-way ANOVA, interaction of strain × condition, *p* = 0.0727, main effect of strain, *p* = 0.258, main effect of condition, *p* = 0.0002). *AVPR1a* mRNA is not altered by S or D in either B6 or BTBR mice (2-way ANOVA, interaction of strain × condition, ns *p* = 0.3335, main effect of strain, ns *p* = 0.6085, main effect of condition, ns *p* = 0.2424). In the BnST, *c-fos* mRNA increases with S (*p* = 0.0003) but not with D (*p* = 0.4869) in B6 and BTBR mice (2-way ANOVA, interaction of strain × condition, *p* = 0.0008, main effect of strain, *p* = 0.3542, main effect of condition, *p* < 0.0001). *OXTR* mRNA increases with S (*p* = 0.0001) or D (*p* = 0.0138) in B6 mice, while it is not altered by either S or D in BTBR mice (2-way ANOVA, interaction of strain × condition, *p* = 0.0211, main effect of strain, *p* = 0.1229, main effect of condition, *p* = 0.0096). *AVPR1a* mRNA decreases with D (*p* = 0.0226) in both B6 and BTBR mice (2-way ANOVA, interaction of strain × condition, *p* = 0.3593, main effect of strain, *p* = 0.0002, main effect of condition, *p* = 0.0005). In the septum, *c-fos* mRNA increases with S (*p* < 0.0001) but not with D (*p* = 0.1526) in B6 and BTBR mice (2-way ANOVA, interaction of strain × condition, *p* = 0.4392, main effect of strain, *p* = 0.9381, main effect of condition, *p* <0.0001). *OXTR* mRNA induction is higher in BTBR mice than B6 mice (2-way ANOVA, interaction of strain × condition, *p* = 0.5441, main effect of strain, *p* < 0.0001, main effect of condition, *p* = 0.1021). *AVPR1a* mRNA increases with S (*p* = 0.0006) in both B6 and BTBR mice (2-way ANOVA, interaction of strain × condition, *p* = 0.6726, main effect of strain, *p* = 0.2618, main effect of condition, *p* = 0.0007). * difference between conditions compared with Controls, *p* < 0.05. n.s., Not significant.

To validate OXT circuitry reaction patterns, we conducted quantitative polymerase chain reaction (qPCR) analyses on selected brain regions of B6 and BTBR mice exposed to either a social stimulus (a novel same-sex mouse) or a chemogenetic activation of OXT^PVN^ neurons (OXT-PVN)(Fig. 5). We analyzed genes that are relevant to OXT neuronal activation via the PVN circuit, including *c-fos* (as a neural activity marker), *OXT*, OXT receptors (*OXTR*), vasopressin (*AVP*), and vasopressin 1a receptors (*AVPR1a*) in the PVN and its projecting regions, MeA, BnST, and septum.

In both B6 and BTBR mice, an increase in *c-fos* mRNA expression was observed in all selected regions following a social encounter, and in the PVN by chemogenetic OXT^PVN^ activation. Social encounter induced *OXT* gene expression in the PVN of both strains and *OXTR* mRNA in the MeA and BnST of B6 mice, but not of BTBR mice. In addition, the social encounter increased *AVPR1a* gene expression in the septum of B6 and BTBR mice. Chemogenetic activation of OXT^PVN^ neurons downregulated *OXT* gene expressions in the PVN and *AVPR1a* expressions in the BnST of both strains. The chemogenetic OXT^PVN^ activation also upregulated *OXTR* expressions in the MeA of both strains, while in the BnST, changes in the *OXTR* mRNA expression (upregulation) were only observed in B6 mice but not in BTBR mice, which represents only strain difference in mRNA expression pattern following a chemogenetic activation examined in the present study.

## Discussion

### OXT circuit-dependent regulation of socio-emotional behavior

Hypothalamic neuronal network plays a predominant role in valence processing of socio-emotional behaviors [43–44]. OXT neurons in the PVN are responsive to dynamic control of approach/avoidance behaviors in social and non-social contexts with a circuit-dependent fashion. We provide strong evidence that the OXT^PVN^→^MeA^ circuit activates social approach behavior, along with anxiogenic profile, while the OXT^PVN^→^BnST^ circuit inhibits social approach, accompanied by anxiolytic response. Social encounter with an unfamiliar counterpart induces c-Fos across the rostral-to-caudal portions of the PVN and its projecting regions, including the BnST (only anterior portion) and MeA, in which the induction of OXT receptor genes was also evident. Chemogenetic stimulation of OXT^PVN^ neurons that concurrently activates both OXT^PVN^→^BnST^ neurons and OXT^PVN^→^MeA^ neurons upregulates the OXT receptor gene expression in both the BnST and MeA; however, suppresses social approach behavior. Therefore, OXT^PVN^→^BnST^ signal that suppresses social approach is significantly effective than OXT^PVN^→^MeA^ signal that generally facilitates social approach when both circuits are simultaneously activated. Social encounter per se induces approach behavior accompanied by the upregulation of OXT receptor gene expression and c-Fos in the MeA and anterior BnST; thus, the OXT^PVN^→^MeA^ signal takes priority to control their behaviors over those OXT^PVN^→^BnST^ signal in a prosocial context. A possible key determinant for OXT circuit balance would act by an inhibitory mediator in the downstream of OXT pathway in the BnST. The posterior BnST and MeA are anatomically characterized as a set of interneuronal nuclei, called as the extended amygdala [45]. It is likely that certain interneuronal connection relayed between the BnST and MeA acts to adjust output balance of neural signals from investigatory approach to non-social stimuli (i.e., OXT^PVN^→^BnST^ signal) to craving approach to prosocial stimuli (i.e., OXT^PVN^→^MeA^ signal).

Accordingly, the MeA–posterior BNST circuits projecting to the hypothalamus are responsible for innate social and predator-defense behaviors [46–47]. Further research is needed to elucidate whether molecular pathway of the OXT circuits maintains balancing of their output within the PVN interneuronal connections or across the inter-regional connections, such as the MeA-posterior BnST circuits.

### Defect of OXT neural circuits accompanied by social deficits in an ASD mouse model

BTBR mice exhibit typical behavioral profile resembling the characteristics of human ASD, including impaired prosocial interaction and persistent, repetitive behaviors [25–26]. Although a target neuronal circuit responsible for controlling these social and non-social deficits exhibited by ASD animal models have been investigated for more than a decade, little is known about the circuit mechanism underlying these behavioral impairments. BTBR mice exhibit decreased densities of OXT neurons across the PVN sub-portions. Furthermore, the OXT^PVN^→^BnST^ circuit, which is massively expressed in B6 mice, appears to be defected in BTBR mice. These malformations of OXT circuit would be linked to blunted responses of neuronal activity patterns across the PVN and its projecting regions following a social encounter. While there is certain dissociation between protein level (e.g., c-Fos IHC) and mRNA level (e.g., *c-fos* qPCR) in response to a social stimulus exposure, the OXT^PVN^→^BnST^ circuit is documented to be defective but the OXT^PVN^→^MeA^ circuit is still intact in BTBR mice.

We recently reported a circuit dysfunction in BTBR mice that shows a compromised circuit in the posterior BnST to the lateral habenula, possibly via attenuating vasopressinergic signal/projection, which contributes to a lack of coordinative responses of social signaling by BTBR mice in a social context [29]. Our present data can extend it to an OXT circuit defect of BTBR mice for controlling prosocial behavior. BTBR mice exhibits agenesis of several brain structures, including the absence of corpus callosum, atrophic of hippocampus, and glial cells overexpression in the cingulate cortex [29, 48]. A MRI imaging analysis revealed that decreased cortical and thalamic grey matter volume along with a reduction of cortical thickness are documented in BTBR mice [49]. It is indicated that one of these morphological mutations occurs in developing brain network of BTBR mice, resulting in a subsided OXT signals and coordinative circuit function that are required to express appropriate approach/avoidance behavior in a social and non-social context.

### Non OXT neurons in the PVN are involved in controlling non-social, defensive components

A pathological repetitive behavior is one of the core symptoms diagnosed as ASD [50]. An underlying mechanism for this exploited behavior would be distinguishable from those for social deficit, although it is still under debate [51–52]. In rodent models, excessive self-grooming and persistent digging/burying the cage bedding materials are considered to recapitulate pathological repetitive behavior states expressed in ASD patients [41]. BTBR mice have repeatedly confirmed these excessive behavioral profiles [25, 33, 53]. Interestingly, nursing with a different, non-ASD, strain can modify social deficits in BTBR mice, but cannot improve their excessive self-grooming [54], suggesting a possibility that different behavioral domains relevant to ASD symptom may be regulated by distinct brain mechanisms. Our data clearly demonstrated that OXT neurons in the PVN are not involved in the responsible mechanism for controlling excessive behavioral profiles, but rather non-OXT neurons in the PVN can stimulate these behaviors including self-grooming and digging/burying cage bedding.

A growing number of pharmacological manipulations on excessive self-grooming behaviors of BTBR mice have fault to archive consistent effects, including the use of cholinergic, glutaminergic, or serotonergic agents [53, 55–57]. Highly complicated, sequential organization of grooming (and related defensive) behaviors raises a hypothesis that various neural circuits regulating discrete components of complex behaviors (e.g., motor, strategy, and sequencing) are involved. Non-OXT PVN neurons offer to facilitate grooming and related obsessive components in both B6 and BTBR mice. The PVN is a multitransmitter nucleus controlling various biological functions [35]. A major stress-related hormone, corticotropin-releasing hormone (CRH) produced in the PVN, are known to induce self-grooming [58–59]. Furthermore, virally induced selective manipulation of vasopressin neurons in the PVN can modify self-grooming in mouse models [60]. These suggest that stress-related, OXT-independent mechanism would be a key mediator for controlling compulsive and defensive components of behavior in the PVN.

## Conclusions

The data demonstrate a circuit-dependent role for OXT signaling in the PVN neurons in controlling social and emotional behaviors and illustrate that the coordinative regulation of two discrete OXT circuits; the OXT^PVN^→^BnST^ and OXT^PVN^→^MeA^ circuits, underlies to control these behaviors. A functional defect of the OXT^PVN^→^BnST^ signal coincides with prosocial behavioral deficits in BTBR mouse model of autism. The study provides novel information about the coordinative role of OXT circuits and establish a foundation for further investigations that delineate the circuit mechanisms of OXT signaling impacting prosocial processes and social deficits relevant to ASD symptom [61].
